# Effects of Achieving SVR on Clinical Characteristics and Surgical Outcomes in Patients Who Developed Early-Stage HCV-Related Hepatocellular Carcinoma and Received Curative Resection: Preoperative versus Postoperative SVR

**DOI:** 10.3390/v14112412

**Published:** 2022-10-31

**Authors:** Po-Yao Hsu, Po-Cheng Liang, Ching-I Huang, Meng-Hsuan Hsieh, Yi-Shan Tsai, Tzu-Chun Lin, Ming-Lun Yeh, Chung-Feng Huang, Chih-Wen Wang, Tyng-Yuan Jang, Yi-Hung Lin, Zu-Yau Lin, Wan-Long Chuang, Chia-Yen Dai

**Affiliations:** 1 Hepatobiliary Division, Department of Internal Medicine, Kaohsiung Medical University Hospital, Kaohsiung Medical University, Kaohsiung 807, Taiwan; 2 Faculty of Internal Medicine, College of Medicine, Kaohsiung Medical University, Kaohsiung 807, Taiwan; 3 Department of Occupational Medicine, Kaohsiung Medical University Hospital, Kaohsiung Medical University, Kaohsiung 807, Taiwan; 4 College of Medicine, National Sun Yat-sen University, Kaohsiung 807, Taiwan; 5 College of Professional Studies, National Pingtung University of Science and Technology, Pingtung 912, Taiwan; 6 Drug Development and Value Creation Research Center, Kaohsiung Medical University, Kaohsiung 807, Taiwan

**Keywords:** chronic hepatitis C, viremia, sustained virologic response, hepatocellular carcinoma, recurrence

## Abstract

The high accessibility to healthcare and increasing awareness of hepatocellular carcinoma (HCC) surveillance after sustained virologic response (SVR) to HCV treatment allow early detection of operable HCC in Taiwan. However, the effects of achieving SVR on patient characteristics and surgical outcomes after curative resection remain elusive. We aimed to compare the clinical presentation and postoperative prognosis among patients with early-stage HCV-related HCC and different viral status. We retrospectively analyzed 208 patients with BCLC stage 0 or A-HCC, including 44 patients who remained HCV viremic, 90 patients who developed HCC after achieving SVR (post-SVR HCC), and 74 patients who subsequently achieved SVR after resection. Patients with post-SVR HCC had a lower degree of hepatitis and better liver function than those who achieved SVR or remained viremic after resection. Notably, 75.6% of patients with post-SVR HCC did not have cirrhosis. Patients with post-SVR HCC and those achieving SVR after resection exhibited comparable recurrence rates and recurrence-free survival, while patients with persistent viremia had the worst surgical outcomes. We concluded that patients with post-SVR HCC had a better liver function but similar surgical outcomes compared with patients who achieved SVR after resection. The low prevalence of cirrhosis in patients with post-SVR HCC highlights the importance of regular surveillance after SVR.

## 1. Introduction

Hepatocellular carcinoma (HCC) is the third most common cause of cancer-related death and ranks sixth in terms of incident cases globally [1]. The majority of HCC arises from underlying liver diseases, such as hepatitis C virus (HCV) or hepatitis B virus (HBV) infection, alcohol abuse, and nonalcoholic fatty liver disease. Taiwan is an epidemic region for HCV, with a much higher prevalence (3.28% [1.8–5.5%]) than other regions of the world, leading to a high incidence rate of HCC [2,3,4]. 

Although interferon (IFN)-based regimens have demonstrated their beneficial effects on recurrence and mortality in patients with HCV-related HCC who underwent curative treatment [5,6,7], ineligibility and treatment-related adverse events often limit the applicability of IFN-based treatment and cause a large gap between clinical efficacy and community effectiveness [2]. Fortunately, these dilemmas have been resolved with the introduction of novel IFN-free, direct-acting antiviral (DAA) agents, which have excellent safety and effectiveness and require a shorter treatment duration. While some previous studies reported unexpectedly high rates of HCC occurrence and recurrence with DAA treatment [8,9,10], subsequent prospective cohort studies and meta-analyses refuted these findings [11,12,13]. 

To eliminate HCV, the National Health Insurance of Taiwan has reimbursed IFN-based therapy since 2003 and DAA therapy since 2017. While more and more HCV-infected patients achieved sustained virologic response (SVR) and gained benefits from HCV eradication, the risk of HCC development persists, especially for those with HCC-related risk factors, such as diabetes, cirrhosis, and high alpha-fetoprotein (AFP) [14,15,16]. With the high accessibility of healthcare in Taiwan, regular surveillance after SVR allows early detection and curative treatment for HCC. However, little is known about the clinical presentation and treatment outcomes after curative therapies in patients who developed early-stage HCC after achieving SVR. It is also unclear if there are different patient and tumor features, recurrence rates after surgical intervention, and recurrence-free survival between patients who developed HCC after SVR and those who achieved SVR after HCC occurrence. Because patient and tumor characteristics play vital roles in the long-term outcomes of HCC after resection, it is essential to characterize these factors in these patient populations [17,18,19,20]. The current study aimed to evaluate and compare the clinical characteristics and surgical outcomes among patients with Barcelona Clinic Liver Cancer (BCLC) stage 0 or A-HCC and different viral status.

## 2. Materials and Methods

### 2.1. Study Population 

This retrospective cohort study reviewed patients with HCC between March 2005 and July 2020 at Kaohsiung Medical University Hospital. The diagnosis of HCC was made based on the criteria of the American Association for the Study of Liver Diseases [21]. The diagnosis was also confirmed by an HCC expert group for each patient. The inclusion criteria were as follows: (1) patients diagnosed with only HCV infection based on the presence of hepatitis C antibody and negative hepatitis B surface antigen (HBsAg); (2) HCC in BCLC stage 0 or A; (3) receiving curative liver resection. We excluded patients who had positive surgical margins, human immunodeficiency virus (HIV) infection, or were concurrent with other cancers. Informed consent was obtained from all individual participants included in the study.

All patients enrolled in this study were categorized into three groups according to the presence of HCV viremia at the time of HCC occurrence and the presence of SVR to HCV treatment: HCC with persistent viremia (patients who had positive HCV RNA at HCC diagnosis and were untreated or failed to achieve SVR), Post-SVR HCC (patients with negative HCV RNA at HCC diagnosis), and viremic HCC with subsequent SVR (patients who achieved HCV SVR after surgical resection). SVR was defined as persistent undetectable serum HCV RNA (24 weeks for patients treated with IFN-based therapy or 12 weeks for patients treated with DAA therapy after the end of treatment). 

### 2.2. Data Collection

All data were obtained retrospectively from the medical record, including age, sex, body mass index (BMI), presence of type 2 diabetes mellitus, hypertension, alcohol drinking, smoking history, serum biochemistry, and AFP. The histological features of the resected tumor, including tumor differentiation, number and size of the tumors, microvascular invasion, capsule invasion, and satellite nodules, were recorded. The presence of hepatic steatosis, Ishak fibrosis score, and Scheuer score were assessed and recorded from non-tumorous liver parts [22,23]. Hepatic steatosis was defined as the presence of ≥5% steatotic hepatocytes in histology specimen. Liver cirrhosis was defined as Ishak fibrosis score 5–6 or Scheuer score 4. 

### 2.3. Surgical Outcome Assessment

All the patients were under regular surveillance for tumor recurrence by sonography, dynamic computed tomography, and/or magnetic resonance imaging every 3–4 months. Recurrence of the tumor was defined as the appearance of new HCC nodules, local tumor progression, or both. Recurrence rates were calculated as the time from surgical resection to recurrence events. Recurrence-free survival (RFS) was defined as the time from surgical resection to HCC recurrence or death from any cause. The census of survival and recurrence status was checked by the end of August 2022. We compared the recurrence rates and RFS between patients in different groups.

### 2.4. Statistical Analysis

Continuous variables were expressed as means ± standard deviations, and categorical variables were presented as number (percentage). The one-way ANOVA was used to compare continuous variables, and the chi square test or Fisher’s exact test was used to compare categorical variables of different groups. The Kaplan–Meier method and the log-rank test were used to analyze recurrence and survival rates. Multivariate analysis with Cox proportional hazard analysis was used to identify the independent prognostic factors. Variables with a potential relationship (*p* < 0.1) identified in the univariate analyses were included in the multivariate analysis. All p values are two-sided, and *p* < 0.05 is determined as statistically significant difference. All database processing and analyses were conducted with SPSS version 24.0 (SPSS, Inc., Chicago, IL, USA).

## 3. Results

### 3.1. Baseline Patient Characteristics

**Figure 1:**
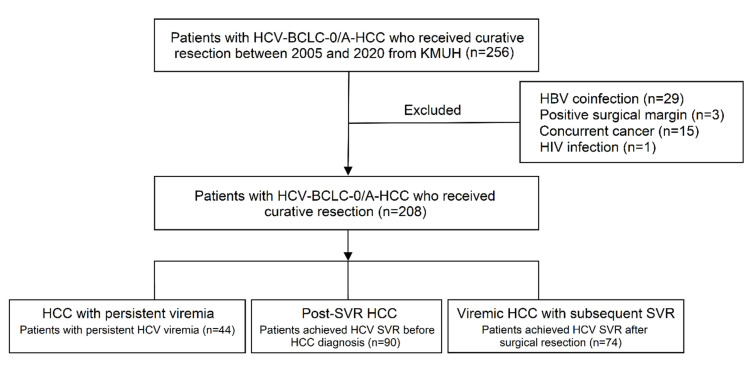
Flow chart of the study.

Between March 2005 and July 2020, 256 patients with BCLC stage 0 or A-HCC who received primary curative hepatectomy were reviewed. We excluded 29 patients with positive HBsAg, three patients who had positive surgical margins, fifteen patients who possessed concurrent cancers other than HCC, and 1 patient who had HIV infection. A total of 208 patients were enrolled in the current study. Forty-four patients had HCC with persistent HCV viremia, 90 patients possessed post-SVR HCC, and 74 patients had viremic HCC and subsequently achieved SVR to antiviral therapies (Figure 1). Their baseline patient characteristics are presented in Table 1. Patients with post-SVR HCC had significantly lower aspartate aminotransferase (AST) levels, lower alanine aminotransferase (ALT) levels, and a higher prevalence of albumin-bilirubin (ALBI) grade I compared to those who had viremic HCC. AFP levels and the presence of histological features, including tumor differentiation, number and size of the tumors, microvascular invasion, capsule invasion, satellite nodules, hepatic steatosis, and cirrhosis, were similar among the three groups.

### 3.2. Surgical Outcomes of HCC with Different Viral Status

**Figure 2:**
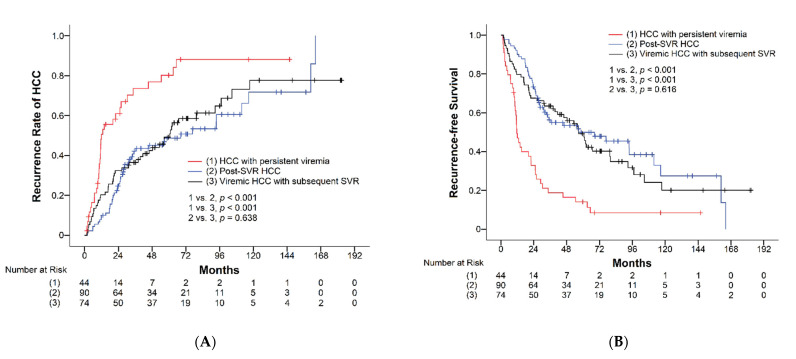
(**A**) Cumulative recurrence rates and (**B**) recurrence-free survival of HCV-related HCC based on viral status.

**Figure 3:**
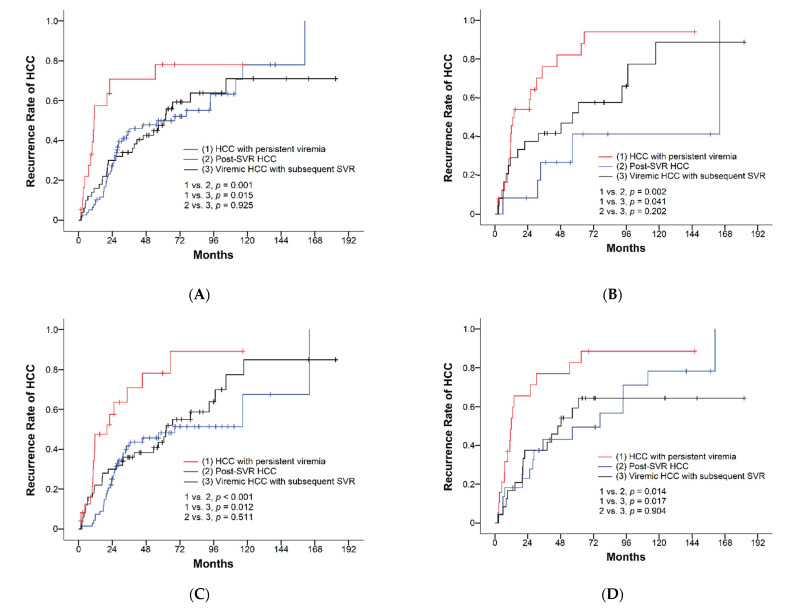
Comparisons of recurrence rates of HCC based on viral status in patients with (**A**) ALBI grade I, (**B**) ALBI grade II/III, (**C**) non-LC, and (**D**) LC.

**Figure 4:**
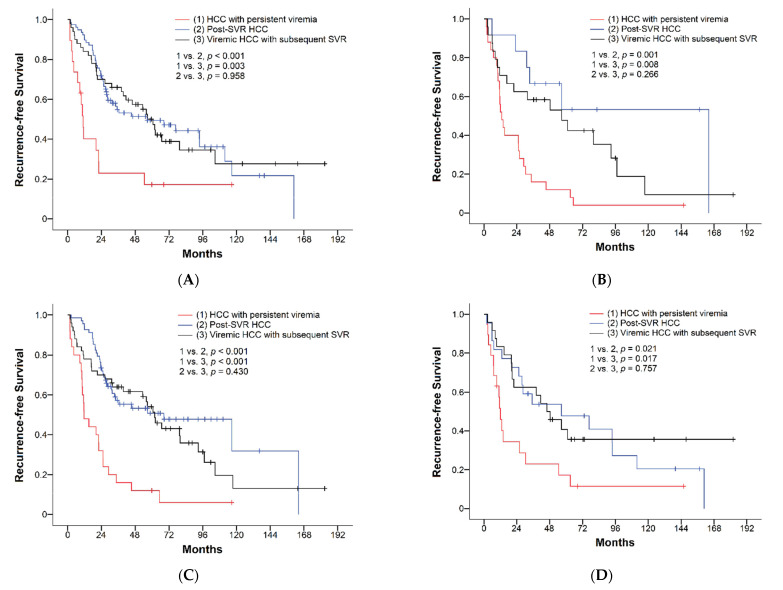
Comparisons of recurrence-free survival of HCC based on viral status in patients with (**A**) ALBI grade I, (**B**) ALBI grade II/III, (**C**) non-LC, and (**D**) LC.

Of the 208 patients with HCC after curative resection, 126 patients (60.6%) developed recurrence during a mean follow-up time of 70.7 months. The cumulative recurrence rates of HCC at the end of the first, third, and fifth year were 35%, 50%, and 61%, respectively (Table 2). Recurrence rates were compared among the three groups: one-year recurrence rates were 60%, 25%, and 32%, three-year recurrence rates were 76%, 45%, and 42%, and five-year recurrence rates were 89%, 51%, and 59% in the HCC with the persistent viremia group, post-SVR HCC group, and viremic HCC with subsequent SVR group, respectively. The recurrence rates of patients achieving SVR either before or after HCC occurrence were significantly lower than that in patients who remained viremic, while the recurrence rates were similar between patients with post-SVR HCC and those achieving SVR after surgical intervention (*p* = 0.638, Figure 2A). During a mean follow-up duration of 70.7 months, the median RFS was 39.5 months (95% CI, 23.1–55.9). The median RFS of the HCC with persistent viremia group, post-SVR HCC group, and viremic HCC with subsequent SVR group were 11.6 months (95% CI, 9.5–13.7), 56.7 months (95% CI, 11.8–101.6), and 56.7 months (95% CI, 43.3–70.0), respectively (Table 2). Patients achieving SVR showed significantly longer RFS than those with persistent viremia (*p* < 0.001), while subjects with post-SVR HCC and those with HCC and subsequent SVR showed comparable RFS (*p* = 0.616, Figure 2B). In subgroup analyses based on various clinical characteristics, similar findings were observed for recurrence rates and RFS (Figure 3A–D and Figure 4A–D). 

### 3.3. Prognostic Factors 

We evaluated the prognostic factors associated with HCC recurrence and RFS. By multivariate analysis, post-SVR HCC (HR, 0.42; *p* < 0.001) and viremic HCC with subsequent SVR (HR, 0.41; *p* < 0.001) were independent factors predictive of lower recurrence rates (vs. HCC with persistent viremia [Table 3]). Post-SVR HCC (vs. HCC with persistent viremia, HR, 0.35; *p* < 0.001), viremic HCC with subsequent SVR (vs. HCC with persistent viremia, HR, 0.34; *p* < 0.001) were independent factors related to longer RFS (Table 4). 

## 4. Discussion

The current study demonstrated that viral status at the time of and after HCC diagnosis is the most important factor influencing patient characteristics and postoperative prognosis. Patients with post-SVR HCC had a lower degree of hepatitis and better liver function than those who had subsequent SVR or remained viremic after HCC diagnosis. Patients with post-SVR HCC and those achieving SVR after resection exhibited comparable recurrence rates and RFS, which were markedly better than those in patients who remained viremic after resection. In multivariate analyses, achieving SVR both before and after HCC occurrence was a substantial factor predictive of lower HCC recurrence and longer RFS compared with constant HCV viremia. 

In our study, lower levels of liver enzymes, a higher proportion of ALBI grade I, higher platelet counts, and a numerically lower incidence of liver cirrhosis in patients achieving SVR before HCC diagnosis indicated that HCV clearance improved hepatitis, leading to a lower degree of liver fibrosis and incidence of cirrhosis as well as a better liver function. These findings were consistent with the results of prior studies [24,25,26,27]. Notably, as many as 75.6% of patients who developed HCC after achieving SVR did not have cirrhosis. This phenomenon might be ascribed to the epigenetic alterations caused by HCV infection, which induce long-term oncogenic effects even after viral eradication [28,29]. Our result highlights the importance of post-SVR surveillance, regardless of the presence of cirrhosis. 

Previous studies mainly focused on the effect of anti-HCV therapy after HCC developed and showed improved liver-related and overall survival [30,31,32]. However, there were limited data regarding the clinical outcomes of patients who developed HCC after achieving SVR. Our prior large multinational study demonstrated that patients with post-SVR HCC had better overall survival (OS) than patients with viremic HCC, while eradicating hepatitis C after HCC occurrence also improved survival. The subgroup analysis for patients with BCLC stage 0/A showed a comparable OS between the post-SVR HCC and the viremic HCC with subsequent SVR groups [25]. With many efforts in managing HCV, including outreach screening and treatment programs for people in hyperendemic areas, patients undergoing dialysis, people who inject drugs, and prisoners, as well as the increasing awareness of HCC surveillance after SVR in Taiwan [33,34,35], we can expect a growing number of patients with operable HCC. The current study expands on the clinical characteristics and surgical outcomes in this population. The current study investigated the recurrence rates and RFS after resection of HCC with various viral status. Similar recurrence rates and RFS were observed between subjects with post-SVR HCC and those with viremic HCC and subsequent SVR. At the same time, patients with persistent viremia had worse surgical outcomes. One multicenter study including 504 Japanese patients who had HCV-related HCC and underwent curative resection exhibited significantly better survival outcomes in patients achieving SVR either before or after hepatectomy than those without SVR [27]. Another study from Japan showed that achieving SVR before HCC development allowed a superior clinical outcome after curative ablation in HCV-related HCC patients [36]. Our results were in line with these studies and further demonstrated similar surgical outcomes between patients with preoperative SVR and those with postoperative SVR.

Considering the potential impact of cirrhosis and liver functional reserve on the surgical outcomes of early-stage HCC, we compared the recurrence rates and RFS in various subgroups. Similar patterns of recurrent rates and RFS were found in all subgroups, including patients with/without cirrhosis and people with different ALBI grades. The similar surgical outcomes and better hepatic functional reserve observed in patients with post-SVR HCC compared with those achieving SVR after resection suggested that achieving SVR might be a stronger factor predictive of lower recurrence rates and longer RFS than liver function. This perspective was supported by the results of multivariate analyses, which exhibited that achieving SVR either before or after HCC was an independent factor associated with recurrence and RFS, while ALBI grade was not significantly associated with surgical outcomes. Previous studies have demonstrated a suboptimal reduction in the risk of disease progression after achieving HCV SVR in patients with decompensated cirrhosis [37,38]. It is unclear if different HCV viral status can result in different prognoses after resection in this population. As only two patients (0.96%) had decompensated cirrhosis (Child–Pugh class B) in this study, it is impossible to address this issue with our data. Further investigation is warranted. 

The current study has some limitations. First, owing to the single-center and retrospective design, a relatively small number of patients, possible selection bias, and unknown confounders that influence surgical outcomes may exist, limiting the applicability of our study’s results to a broader population. Because this is the first study to compare the surgical outcomes after curative resection between post-SVR HCC and viremic HCC with subsequent SVR, more investigations are needed to verify our findings. Second, we failed to exclude patients with occult or past HBV infection due to a lack of data on anti-HBc antibody and HBV-DNA. Occult or past HBV infection can be prevalent in an HBV-endemic country such as Taiwan [39]. An increasing number of studies have reported more advanced tumor histological grades in patients with HCV and occult HBV infection (OBI) compared with patients without OBI, potentially influencing the postoperative prognosis [40]. Additionally, we did not analyze the interval between achieving SVR and hepatectomy. Previous studies have shown that a longer interval between achieving SVR and curative therapies was associated with better surgical outcomes [36,41].

## 5. Conclusions

In conclusion, our results demonstrated that patients with post-SVR HCC had a better liver function but similar clinical outcomes after curative resection compared with patients who achieved SVR after resection. Achievement of SVR both before and after HCC occurrence, in comparison to persistent HCV viremia, was the most important factor associated with lower recurrence rates and longer RFS. Our data highlight the beneficial impact on liver function and clinical outcomes as well as the necessity of timely antiviral treatment for HCV-infected patients. In addition, given that 75.6% of patients with post-SVR HCC did not have cirrhosis, HCC surveillance after SVR should be individualized based on the degrees of liver fibrosis and the presence of HCC-related risk factors instead of restricted to cirrhotic patients [42].

## Figures and Tables

**Table 1:** Baseline characteristics of the 208 patients with early-stage HCV-related HCC.

Variable	All Patients (n = 208)	HCC with Persistent Viremia (n = 44)	Post-SVR HCC (n = 90)	Viremic HCC with Subsequent SVR (n = 74)	*p* Value
Age (years), mean ± SD	64.6 ± 8.6	67.5 ± 8.2	64.7 ± 8.1	62.8 ± 8.9 †	0.016
Male gender, n (%)	136 (65.4)	25 (56.8)	65 (72.2)	46 (62.2)	0.163
BMI (kg/m^2^), mean ± SD	24.6 ± 3.8	24.2 ± 3.5	25.4 ± 4.1	23.9 ± 3.3 ‡	0.032
Diabetes mellitus, n (%)	68 (32.7)	17 (38.6)	32 (35.6)	19 (25.7)	0.260
Hypertension, n (%)	104 (50.0)	25 (56.8)	54 (60.0)	25 (33.8) †‡	0.002
Alcohol drinking, n (%)	44 (21.2)	8 (18.2)	22 (24.4)	14 (18.9)	0.595
Smoking, n (%)	61 (29.3)	9 (20.5)	29 (32.2)	23 (31.1)	0.342
AST (IU/L), mean ± SD	53.6 ± 44.2	68.3 ± 28.8	37.9 ± 52.1 †	64.0 ± 34.6 ‡	<0.001
ALT (IU/L), mean ± SD	55.2 ± 42.5	68.7 ± 39.3	34.7 ± 32.2 †	72.1 ± 44.9 ‡	<0.001
Platelet count (10^3^/μL), mean ± SD	166.9 ± 71.1	148.0 ± 54.5	181.2 ± 62.9 †	160.8 ± 85.3	0.025
ALBI grade I/II/III					<0.001
Grade I, n (%)	147 (70.7)	19 (43.2)	78 (86.7) †	50 (67.6) †‡	
Grade II, n (%)	59 (28.4)	24 (54.5)	11 (12.2) †	24 (32.4) ‡	
Grade III, n (%)	2 (1.0)	1 (2.3)	1 (1.1)	0 (0)	
AFP ≥ 20 ng/mL, n (%)	80 (38.5)	25 (56.8)	26 (28.9) †	29 (39.2)	0.008
AFP ≥ 200 ng/mL, n (%)	32 (15.4)	11 (25.0)	12 (13.3)	9 (12.2)	0.135
BCLC stage 0/A, n (%)	71/137 (34.1/65.9)	13/31 (29.5/70.5)	31/59 (34.4/65.6)	27/47 (36.5/63.5)	0.742
Histological grade					0.168
Well-differentiated, n (%)	27 (13.0)	7 (15.9)	7 (7.8)	13 (17.6)	
Moderately differentiated, n (%)	144 (69.2)	33 (75.0)	64 (71.1)	47 (63.5)	
Poorly differentiated, n (%)	37 (17.8)	4 (9.1)	19 (21.1)	14 (18.9)	
Multiple tumors, n (%)	19 (9.1)	3 (6.8)	8 (8.9)	8 (10.8)	0.822
Largest tumor size (cm), mean ± SD	2.6 ± 1.1	2.5 ± 1.1	2.5 ± 1.0	2.6 ± 1.2	0.832
Microvascular invasion, n (%)	43 (20.7)	9 (20.5)	20 (22.2)	14 (18.9)	0.873
Capsule invasion, n (%)	75 (36.1)	13 (29.5)	32 (35.6)	30 (40.5)	0.481
Satellite nodules, n (%)	52 (25.0)	14 (31.8)	19 (21.1)	19 (25.7)	0.400
Hepatic steatosis, n (%)	100 (48.1)	26 (59.1)	46 (51.1)	28 (37.8)	0.061
Liver cirrhosis, n (%) ¶	65 (31.3)	19 (43.2)	22 (24.4)	24 (32.4)	0.086
Follow-up (months), mean ± SD	70.7 ± 43.3	50.5 ± 37.6	67.0 ± 40.4	87.3 ± 44.2 †‡	<0.001

Abbreviations: BMI, body mass index; SVR, sustained virologic response; IFN, interferon; DAA, direct-acting antiviral; AST, aspartate aminotransferase; ALT, alanine aminotransferase; ALBI, Albumin-bilirubin; AFP, alpha-fetoprotein; BCLC, Barcelona Clinic Liver Cancer. Continuous data were presented as mean ± standard deviation; Categorical data were presented as number (%). † *p* < 0.05 vs. HCC with persistent viremia; ‡ *p* < 0.05 vs. post-SVR HCC; ¶ Liver cirrhosis was defined as Ishak fibrosis score 5–6 or Scheuer score 4 from non-tumor part.

**Table 2:** Prognosis of the 208 patients with HCV-related early-stage HCC based on HCV status.

	All Patients (n = 208)	HCC with Persistent Viremia (n = 44)	Post-SVR HCC (n = 90)	Viremic HCC with Subsequent SVR (n = 74)	HCC with Persistent Viremia vs. Post-SVR HCC *p* Value	HCC with Persistent Viremia vs. Viremic HCC with Subsequent SVR *p* Value	Post-SVR HCC vs. Viremic HCC with Subsequent SVR *p* Value
Cumulative recurrence rates					<0.001	<0.001	0.638
1-year recurrence rate	35%	60%	25%	32%			
3-year recurrence rate	50%	76%	45%	42%			
5-year recurrence rate	62%	89%	51%	59%			
Recurrence-free survival, median (RFS), months (95% CI)	39.5 (23.1–55.9)	11.6 (9.5–13.7)	56.7 (11.8–101.6)	56.7 (43.3–70.0)	<0.001	<0.001	0.616
1-year RFS	63%	33%	73%	68%			
3-year RFS	47%	16%	53%	58%			
5-year RFS	36%	8%	48%	40%			

Abbreviations: SVR, sustained virologic response; CI, confidence interval; RFS, recurrence-free survival.

**Table 3:** Factors predictive of HCC recurrence.

	Univariate		Multivariate	
Predictor	HR (95% CI)	*p* Value	HR (95% CI)	*p* Value
Age ≥ 60 (years)	1.38 (0.92–2.07)	0.126		
Male	1.11 (0.77–1.61)	0.569		
Diabetes mellitus	1.25 (0.86–1.80)	0.243		
Hypertension	0.84 (0.59–1.19)	0.332		
Alcohol drinking	0.90 (0.58–1.39)	0.619		
Smoking	0.79 (0.53–1.18)	0.244		
AST ≥ 40 (IU/L)	1.31 (0.92–1.86)	0.138		
ALT ≥ 40 (IU/L)	1.36 (0.96–1.94)	0.085	1.21 (0.81–1.80)	0.358
Platelet ≥ 150 (10^3^/μL)	0.72 (0.50–1.02)	0.064	0.72 (0.50–1.05)	0.089
ALBI grade II/III (vs. grade I)	1.26 (0.86–1.83)	0.233		
AFP ≥ 200 ng/mL	1.44 (0.90–2.31)	0.127		
HCV status				
HCC with persistent viremia		Reference		Reference
Post-SVR HCC	0.37 (0.24–0.58)	<0.001	0.42 (0.26–0.68)	<0.001
Viremic HCC with subsequent SVR	0.41 (0.26–0.65)	<0.001	0.41 (0.26–0.66)	<0.001
BCLC stage A (vs. stage 0)	1.41 (0.96–2.06)	0.079	1.25 (0.74–2.11)	0.413
Multiple tumors	1.04 (0.59–1.85)	0.886		
Largest tumor size (cm)	1.16 (0.99–1.37)	0.065	1.11 (0.89–1.38)	0.377
Histological grade: poor/moderate (vs. well)	1.40 (0.81–2.45)	0.232		
Microvascular invasion	1.14 (0.74–1.77)	0.548		
Capsule invasion	1.12 (0.78–1.61)	0.538		
Satellite nodules	1.53 (1.04–2.26)	0.031	1.59 (1.06–2.38)	0.025
Hepatic steatosis	1.38 (0.97–1.96)	0.072	1.41 (0.97–2.04)	0.070
Liver cirrhosis †	1.30 (0.90-1.87)	0.159		

Abbreviations: HR, hazard ratio; CI, confidence interval; AST, aspartate aminotransferase; ALT, alanine aminotransferase; ALBI, albumin-bilirubin; AFP, alpha-fetoprotein; SVR, sustained virologic response; BCLC, Barcelona Clinic Liver Cancer. † Liver cirrhosis was defined as Ishak fibrosis score 5–6 or Scheuer score 4 from non-tumor part.

**Table 4:** Factors predictive of recurrence-free survival.

	Univariate		Multivariate	
Predictor	HR (95% CI)	p Value	HR (95% CI)	*p* Value
Age ≥ 60 (years)	1.40 (0.95–2.08)	0.093	1.31 (0.86–1.99)	0.214
Male	1.06 (0.74–1.50)	0.756		
Diabetes mellitus	1.23 (0.86–1.76)	0.257		
Hypertension	0.83 (0.59–1.16)	0.263		
Alcohol drinking	0.89 (0.59–1.36)	0.601		
Smoking	0.77 (0.52–1.13)	0.184		
AST ≥ 40 (IU/L)	1.33 (0.94–1.86)	0.104		
ALT ≥ 40 (IU/L)	1.35 (0.96–1.89)	0.085	1.25 (0.84–1.85)	0.275
Platelet ≥ 150 (10^3^/μL)	0.71 (0.51–0.99)	0.049	0.71 (0.49–1.04)	0.077
ALBI grade II/III (vs. grade I)	1.38 (0.97–1.96)	0.077	0.88 (0.58–1.34)	0.560
AFP ≥ 200 ng/mL	1.48 (0.94–2.32)	0.091	1.59 (1.00–2.54)	0.051
HCV status				
HCC with persistent viremia		Reference		Reference
Post-SVR HCC	0.32 (0.21–0.49)	<0.001	0.35 (0.21–0.57)	<0.001
Viremic HCC with subsequent SVR	0.36 (0.23–0.56)	<0.001	0.34 (0.21–0.54)	<0.001
BCLC stage A (vs. stage 0)	1.46 (1.01–2.11)	0.046	1.25 (0.75–2.08)	0.392
Multiple tumors	1.05 (0.60–1.82)	0.877		
Largest tumor size (cm)	1.17 (1.00–1.37)	0.046	1.10 (0.89–1.37)	0.370
Histological grade: poor/moderate (vs. well)	1.42 (0.83–2.42)	0.204		
Microvascular invasion	1.25 (0.83–1.89)	0.286		
Capsule invasion	1.09 (0.77–1.55)	0.616		
Satellite nodules	1.48 (1.02–2.16)	0.040	1.43 (0.97–2.11)	0.069
Hepatic steatosis	1.29 (0.92–1.81)	0.141		
Liver cirrhosis †	1.20 (0.84–1.71)	0.318		

Abbreviations: HR, hazard ratio; CI, confidence interval; AST, aspartate aminotransferase; ALT, alanine aminotransferase; ALBI, albumin-bilirubin; AFP, alpha-fetoprotein; SVR, sustained virologic response; BCLC, Barcelona Clinic Liver Cancer. † Liver cirrhosis was defined as Ishak fibrosis score 5–6 or Scheuer score 4 from non-tumor part.

## Data Availability

The data that support the findings of this study are not publicly available due to their containing information that could compromise the privacy of research participants but are available from the corresponding author [C.-Y.D.] upon reasonable request.
